# 

**Published:** 1891-01-31

**Authors:** 


PRICE ONE PENNY.

[N]o. 227, Vol. IX.—January 31st, 1891.

[Registered at the General Post Office as a Newspaper for transmission in the United Kingdom and Abroad.]

WITH EXTRA NURSING SUPPLEMENT.

Per Annum, 6s. 6d., post free.

Monthly Parts, 6d.; post free 8d.

Ditto per Annum, 8s., post free.

### THE HOSPITAL

Glasgow Western Infirmary — St. Thomas's Hospital — Norfolk and Norwich Hospital

A WEEKLY JOURNAL OF

**Science, Medicine, Nursing and Philanthropy.**

SATURDAY, JANUARY 31st, 1891.

**Are Doctors so very Bad?** . . . 271
**Passing Topics.**—A Mild Rebuke . . . 272
**The Doctor's Armchair.**—Science in Straits . . . 273
**The Microscope in Medicine.** By Frank J. Wethered, M.D.—III. The Microtome and Hardening Specimens . . . 274
**The London Poor** . . . 275
**The Metropolitan Hospital Saturday Fund** . . . 275
**Notes and News** . . . 276
**Scraps and Gleanings** . . . 277
**Annotations.**—Physical Education—About Breakfast—The Hospitals Commission Again . . . 278
**The Practitioner's Mirror and Retrospect:—**
Medicine.—Rickets: Treatment by Phosphorus—Chorea, 279; The Etiology of Cancer . . . 280
Surgery.—Surgical Treatment of Hydrocephalus—Association of Gangrene and Tetanus . . . 280
Therapeutics.—Strychnine as an Antidote . . . 281
**Editor's Letter-Box.**—Infants' Food—**Books for Review** 281
**Recreation in Relation to the Health of the People.** II.—Beginning at the Bottom . . . 282
**The Student of Medicine.**—Notes from the Schools—Appointments—Vacancies—Pass Lists—Athletics . . .

**EXTRA SUPPLEMENT—THE NURSING MIRROR.**

**En Passant.**—Rural Nursing Association—Perth Nursing Society—A Midwife in Trouble—A gentlemanly Discussion—Keeping Christmas—Short Items—Rising to the Occasion . . . xcv

**Lectures on Surgical Ward Work and Nursing.** By Alex. Miles, M.B. Edin., C.M., F.R.C.S.E.. Lecture XI.—The Spray Dressing (*continued*) . . . xcvi

**Nursing Medals and Certificates**—Kent Nursing Institution . . . xcvii

**Sewing Competitions** . . . xcvii

**Appointments** . . . xcvii

**For Reading to the Sick.**—The Sun . . . xcvii

**Medical Applications of Electricity** (*continued*) . . . xcviii

**Nurses on Their Travels.**—Buenos Ayres . . . xcviii

**Examination Questions** . . . xcix

**Presentations** . . . xcix

**The Plain Path** . . . xcix

**Everybody's Opinion.**—Princess Christian's Daughter . . . xcix

**Notes and Queries** . . . xcix

**The World Below the Diaphragm** . . . c

**What to Read—Impromptu** . . . c

**Amusements and Relaxation** . . . c

